# In memoriam: Sasan Aliniaeifard (1981 – 2025)

**DOI:** 10.3389/fpls.2025.1629009

**Published:** 2025-06-24

**Authors:** Elahe Javadi Asayesh, Mohammadhadi Sobhani, Priscilla Malcolm Matamoros, Tao Li, Leo F. M. Marcelis, Ernst Woltering, Elias Kaiser

**Affiliations:** ^1^ Horticulture and Product Physiology, Department of Plant Sciences, Wageningen University, Wageningen, Netherlands; ^2^ Plant Breeding, Department of Plant Sciences, Wageningen University, Wageningen, Netherlands; ^3^ Phenovation BV, Wageningen, Netherlands; ^4^ Institute of Environment and Sustainable Development in Agriculture, Chinese Academy of Agricultural Sciences, Beijing, China; ^5^ Food and Biobased Research, Wageningen University and Research, Wageningen, Netherlands; ^6^ Research Institute of Agriculture and Life Sciences, Seoul National University, Seoul, Republic of Korea

**Keywords:** stomatal malfunctioning, salinity, ABA, controlled environment agriculture (CEA), photosynthesis

## Abstract

We celebrate the life and mourn the untimely passing of Dr. Sasan Aliniaeifard, an inspiring scientist, teacher, entrepreneur and mentor, who contributed to the science of controlled environment agriculture (CEA) in Iran and beyond.

## Biographical background and key scientific achievements

Sasan Aliniaeifard was born in Khorramabad, Iran, on 22 March, 1981. After studying B.Sc. *Plant production engineering-Horticulture* at Kurdistan University, he obtained his M.Sc. in *Agricultural engineering-Horticulture* at Tabriz University. In 2010, he obtained a scholarship, became a PhD candidate at the *Horticultural Supply Chains* chair group at Wageningen University and Research (WUR), and defended his PhD thesis in 2014. A few days after defending his PhD, he became an assistant professor in the Horticulture department belonging to the Faculty of Agricultural Technology at the University of Tehran (UT). In April 2021, he was promoted to associate professor. At the time of his death, he was in the process to becoming a full professor. He was the recipient of several awards, including prices for excellent education, international collaboration, and research, at UT. In 2023 and 2024, he was named among the world’s top 2% most-cited scientists in his field based on age (Elsevier Data Repository, 2024). His academic contributions include 116 Scopus-indexed publications. At the time of writing, his h-index is 39, while his i10-index is 89, suggesting that his work is well recognized in the research community.

## Ph.D. research at Wageningen University

Stomata usually close when air humidity is low (high vapor pressure deficit, VPD) due to signaling by abscisic acid (ABA). However, when plants are continuously grown under low VPD conditions, they can become insensitive to ABA and thus lose the ability to close. This form of stomatal malfunctioning can severely shorten the post-harvest life of horticultural products, including cut flowers. Sasan took on this topic during his PhD research, and in the process became an expert on stomatal functioning in relation to environmental conditions. He worked with a range of plant species (*Vicia faba*, *Chrysanthemum morifolium*, and *Arabidopsis thaliana*) and experimental techniques (chlorophyll fluorescence imaging under low [O_2_], microscopy, gravimetry, porometry, leaf anatomy analysis, and LC-MS/MS). As a result of his PhD research, four experimental publications (Aliniaeifard and van Meeteren, 2014, 2016; Aliniaeifard et al., 2014; Van Meeteren et al., 2020) and a literature review (Aliniaeifard and van Meeteren, 2013) were published in highly reputable plant science journals (*Journal of Experimental Botany*, *Physiologia Plantarum* and *Scientia Horticulturae*).

During his PhD, he was supervised by Dr. Uulke van Meeteren and Prof. Ernst Woltering, who remember him as a very enthusiastic, ambitious and hard-working researcher with original ideas. Experimental research is often difficult, and every PhD student experiences setbacks, delays and disappointments. On such occasions, it was striking that Sasan kept his good spirits, and immediately repeated the experiment or designed new experiments to prove his case.

At WUR, Sasan built lasting friendships that persisted during the rest of his scientific career. He shared a large office with many other PhD students, with some of whom he had long conversations about research, life and culture. He also guided M.Sc. students with exceptional kindness and patience. As a result of his guidance, one of his M.Sc. students, Priscila Malcolm Matamoros, became the co-author of two publications resulting from his PhD project, which is a rare occurrence.

Importantly, Sasan was not alone during his PhD project: his wife, Dr. Maryam Seifi Kalhor, was working on obtaining her PhD at the same time, in the Laboratory of Molecular Biology at WUR. Maryam’s project focused on root nodule symbiosis between a non-legume, *Parasponia*, and a soil bacterium, *rhizobium*. She was supervised by Dr. Rene Geurts and Prof. Ton Bisseling. Maryam’s and Sasan’s son Avash was born in Wageningen, in the midst of both of their PhD projects.

## Academic and professional roles

After starting work as an assistant professor at UT, Sasan focused on crop yield and physiology under controlled environment conditions and abiotic stress. In Iran, water scarcity affects agriculture, and this is likely to get worse. Sasan foresaw the looming water crisis, and believed that advancing academia was essential to develop greenhouses and vertical farms with minimal water use. He envisioned a future where Iran would rely on CEA for vegetables and even strategic cereals such as wheat and rice, to ensure long-term food security. Sasan decided to tackle this challenge by focusing on introducing modern controlled cultivation methods from the ground up - first to the scientific community and later to farmers, industry professionals and government officials. In 2016, Sasan established the *Photosynthesis and light responses lab*, to conduct research and simultaneously introduce recent approaches in CEA. After securing space at the Department of Horticulture at UT, he equipped it using his own funds. Initial difficulties in obtaining equipment did not stop him: for instance, he travelled to the Czech Republic, bought a chlorophyll fluorescence camera (FluorCam; Photon Systems Instruments), and brought it back home. When it was difficult for him to buy specific LED lamps, he asked his long-time friend and former PhD colleague, Dr. Tao Li, to ship them from China. During the process of establishing the laboratory, he encountered many difficulties, but he tried his best to solve them, demonstrating his determination to conduct science.

Over time, the lab grew. Growth chambers with different light spectra were developed. In 2019, the lab received the distinguished lab award at UT. Important scientific publications focused on environmental stresses (salinity, cadmium, high light intensity), and a number of external and internal factors that could ameliorate the effects of these stresses (γ-Aminobutyric acid (GABA), calcium signaling, flavonoids, and acclimation to different light spectra; Lastochkina et al., 2017; Bayat et al., 2018; Kalhor et al., 2018; Seifikalhor et al., 2019b, 2019a, 2020; Shomali et al., 2022). Importantly, his wife was a co-author in many of these publications, suggesting that Sasan and Maryam were a team in both their professional and private lives. In 2022, Sasan expanded his lab and established the CEA center at the University of Tehran (CEAC-UT). This facility consists of a seedling preparation hall, growth chambers, photosynthesis laboratory, phenotyping facilities, robotic UV application system, and several hydroponic systems. In 2022, he and his wife also co-founded Parcham, a knowledge-based enterprise focused on vertical farming and the use of supplemental LED lighting.

Sasan was fully engaged in academic activities, including translating and authoring books, writing and reviewing scientific papers, and participating in national and international congresses. He was the managing editor and an editorial board member of the *International Journal of Horticultural Science and Technology*. He also played a pioneering role in defining and suggesting new interdisciplinary fields, particularly by integrating plant science and greenhouse structural engineering at UT. As a coordinator and lecturer, he taught courses on plant biochemistry, plant hormones, horticulture, greenhouse management and automation, and physiology of horticultural plants. Also, he taught and organized more than 15 specialized courses/workshops for professionals in the horticultural industry.

## Personal attributes

Sasan’s passion for science was contagious. He always had a smile ready for those he talked to, and inspired his students to take pride in their role of contributing to agriculture and food production. He was well known for his kindness, flexibility, and respectful personality. He often had friendly conversations with his students, discussing topics like football, life and everyday moments, which helped create a warm and approachable atmosphere around him. He dedicated lots of time to his students, often spending long hours in the lab. He was always available for guidance and support.

Beyond his personal ambitions, Sasan demonstrated a strong sense of teamwork and an exceptional capacity for hard work. He was always open to networking, and eager to collaborate with new colleagues. This is reflected in his research projects, many of which were conducted in collaboration with various companies and universities across Iran as well as the Netherlands, China, Russia, Greece and Germany. In his lab, he strived to apply the academic practices and culture he had experienced while at WUR. This meant teaching valuable skills such as presenting with confidence, preparing high-quality presentation slides, and communicating research clearly. He organized English-language discussion groups on scientific papers and topics related to horticulture, thereby encouraging students to improve their English proficiency.

## Legacy

On March 21, 2025—the first day of the Persian New Year (Nowruz) and just one day before his birthday—Sasan tragically lost his life in a car accident, along with his wife and their son, Avash. They are survived by their daughter, Avina. Sasan will be remembered not only as a dedicated and accomplished scientist and teacher, but equally for the kind, cheerful, thoughtful, and warm-hearted person he was. He leaves behind valuable contributions to plant science as well as a large number of former students who continue their academic and professional journeys at universities around the world.
